# Beri-Beri in the Fiji Islands

**Published:** 1896-09

**Authors:** 


### BERI-BERI IN THE FIJI ISLANDS.

A valuable contribution to the natural history of beri-beri is contained in the recently issued official reports on an outbreak of the disease which occurred in the year 1894 in the British colony of Fiji. All the patients were Japanese coolies of whom about three hundred were imported for the purpose of working on sugar plantations. Their ages varied from eighteen to thirty years, and they arrived in the colony on April 27, 1894. It was understood that they were all medically examined in Japan, and on landing in Fiji they were, with few exceptions, a fine body of men; but during the first month of their employment, which was chiefly cane-cutting and draining newly-cleared land, there was a good deal of sickness among them, mostly malarial fever and diarrhœa.

The first hospital case of beri-beri was admitted on May 30th, in the month of July at least seventeen of the men complained that they were suffering from kakke, the ordinary name for beri-beri in Japan, where it is well known to be endemic; and in the middle of November there were more than two hundred and twenty cases. There were in all sixty-eight Japanese known to be attacked, of whom sixty-five died, and in February, 1895, the whole of the survivors were sent back to Japan.

The disease was entirely confined to these emigrants, not spreading either to the Indian laborers who worked on the same plantations with them, or to the Fijian inhabitants of the villages. The Japanese were lodged in well-ventilated houses, all of which (with one exception) were constructed of wood and were spacious enough to allow at le st three hundred and thirty cubic feet to each occupant. After their departure these buildings, as well as the hospitals, were washed with a solution of corrosive sublimate of the strength of one in one thousand, to which strong hydrochloric acid was added. Two days later the buildings were well washed with hot water and soap, and left open for a week, after which the washings with corrosive sublimate and soap and water were repeated. They were then occupied by Indian coolies, among whom no cases of beri-beri appeared during the eleven months that elapsed up to the date of the report.

The colonial medical officers devoted much attention to the relations existing between beri-beri as it appeared in this epidemic and other diseases which either simulate it or have passed under the same name. They conclude that it is independent of scurvy, pernicious anemia,or ankylostomiasis, and that it is a specific neuritis affecting the motor and sensory nerves, peripherial at first, but afterwards involving muscles and vital organs supplied by the sympathetic system. It causes death either by paralyzing the heart and respiratory muscles or by choking the lungs and other viscera with the edematous effusion to which, in one class of cases, it gives rise. The most usual preliminary symptoms were a feeling of heaviness and weariness in the legs, numbness of the calf muscles, slight edema over the crest and inner surface of the tibia, loss of appetite, giddiness, headache, shortness of breath, palpitation, rapid pulse, and digestive disorders. With respect to the motor paralyses, the calf muscles were generally first affected, then the peronei and the flexors of the feet and toes. The muscles of the hands and fingers were usually the next group affected. As a rule the patellar reflex was early impaired, and with the progress of the disease wholly suppressed. Some cases, however, with well-marked motor impairment exhibited good patellar reflexes, and in some the reflex was exaggerated. Cutaneous anesthesia was constantly present over the calves, the ankles, and the dorsa of the feet, accompanied by pain in the deeper parts when firm pressure was made. In a few cases very small rod-shaped bodies were observed in the blood, but cultivation experiments were unsuccessful.—*Boston Medical and Surgical Journal.*

